# Dynamics of balance indicators, activities of daily living, and quality of life of elderly suffering from Parkinson’s disease and frailty after proximal humerus fracture following physiotherapeutic functional training

**DOI:** 10.25122/jml-2021-0386

**Published:** 2022-01

**Authors:** Bohdan Hrytsuliak, Zinovii Ostapiak, Yurii Polataiko, Roman Herych, Bogdan Lisovskyi, Eduard Lapkovskyi, Hanna Karpenko, Liliia Vojchyshyn, Olha Zastavna, Lidiia Sheremeta, Tamara Berezna, Olesia Herych

**Affiliations:** 1. Department of Human and Animal Anatomy and Physiology, Vasyl Stefanyk Precarpathian National University, Ivano-Frankivs’k, Ukraine; 2. Department of Theory and Method of Physical Culture and Sports, Vasyl Stefanyk Precarpathian National University, Ivano-Frankivs’k, Ukraine; 3. Department of Sports-Pedagogical Disciplines, Vasyl Stefanyk Precarpathian National University, Ivano-Frankivs’k, Ukraine; 4. Department of Physical Therapy and Ergotherapy, Faculty of Physical Education and Sports, Vasyl Stefanyk Precarpathian National University, Ivano-Frankivs’k, Ukraine; 5. Department of Foreign Languages, Vasyl Stefanyk Precarpathian National University, Ivano-Frankivs’k, Ukraine; 6. Department of Children’s Diseases, Academic and Research Institute of Postgraduate Education of IFNMU, Ivano-Frankivs’k, Ukraine; 7. Department of Otorhinolaryngology Head and Neck Surgery, Ivano-Frankivsk National Medical University, Ivano-Frankivs’k, Ukraine

**Keywords:** physical therapy, old age, frailty, Parkinson’s disease, rehabilitation, PD – Parkinson’s disease, PHF – proximal humerus fractures, UL – upper limb, SPPB – Short Physical Performance Battery, BBS – Berg Balance Scale, DASH – Disability of the Arm, Shoulder and Hand Outcome Measure, FES-I – Falls efficacy Scale International, ADL Index – Barthel Activities of daily living Index, IADL Scale – Lawton Instrumental activities of daily living Scale, PDQ-39 – Parkinson’s disease Questionnaire-39

## Abstract

Balance dysfunction in elderly patients with Parkinson’s disease (PD) is a high-risk fall precaution, along with sarcopenia and senile asthenia, which leads to traumas, including fractures of the proximal humerus fractures (PHF). The objective of the study was to determine the effectiveness of a functional training as part of a physical therapy program on balance, upper limb (UL) function, daily living activities, and quality of life in elderly patients with PD and frailty, following proximal humerus fractures. We examined 33 elderly patients with PD and frailty in the recovery period after PHF. The control group included individuals who underwent rehabilitation according to the general principles of kinesitherapy. The treatment group consisted of patients engaged in a program of physical therapy directed at improving balance, function of the upper UL, motor stereotype, and activities of daily living. The effectiveness of the program was evaluated using the Short Physical Performance Battery (SPPB), Berg Balance Scale (BBS), Disability of the Arm, Shoulder and Hand Outcome Measure (DASH), Wrist Dynamometry, Falls efficacy Scale International (FES-I), Barthel Activities of daily living (ADL) Index, Lawton Instrumental activities of daily living (IADL) Scale, PD Questionnaire-39 (PDQ-39). According to all studied indicators, the patients of both groups showed a statistically and significantly better result compared to the initial data (p<0.05), but the treatment group showed better outcomes compared with the control group (p<0.05).

## Introduction

Parkinson’s disease is a chronic progressive brain disease, mainly associated with the degeneration of dopaminergic neurons of the substantia nigra, with the accumulation of α-synuclein protein and the formation of Lewy bodies in the neurons, which causes various movement disorders [1]. Along with motor symptoms (hypokinesia, muscle rigidity, resting tremor, and postural instability), patients develop a wide range of non-motor disorders in the form of autonomic dysfunction (orthostatic hypotension, constipation, sweating disorders), memory and intellectual impairment, sleep disorders, decreased sense of smell, seborrhea, pain, neuropsychiatric symptoms (apathy, depression, anxiety, panic attacks etc) [2]. Slowness and discomfort of movements, muscle stiffness, difficulty initiating movements, impaired control over body position, tremor, and involuntary movements lead to impaired motor control, changes in posture, and gait.

Impaired postural stability leads to falls - a common concomitant problem of Parkinson’s disease, which leads to traumatic injuries (dislocations, bruises, fractures etc). Additional risk factors for falls are old age, significant duration of Parkinson’s disease, concomitant neuro-musculoskeletal changes, decreased leg muscle strength, changes in proprioception and gait speed, increased gait variability, “freezing” gait [3, 4]. Another predictor of decline in Parkinson’s disease is an increased risk of osteoporosis. Patients with Parkinson’s disease are almost two times more likely to develop fractures [3, 5]. Levodopa is a common drug for Parkinson’s disease treatment, but it is also associated with an increased risk of fractures. On the one hand, Levodopa might reduce bone mineral density but also increase patient mobility without improving postural stability, allowing patients to move more freely while maintaining a high risk of falls [5].

Fractures of the proximal humerus as an independent pathology rank third in frequency among bone fractures in the elderly, second only to fractures of the proximal femur and distal radial bone [6]. Epidemiological studies indicate a steady increase in the incidence of such fractures and predict a doubling of this number in older patients over the next 20 years [7]. Most proximal humerus fractures in elderly patients occur as a result of low-energy trauma. In the presence of osteoporosis, it is enough to fall on the arm to cause such a fracture compared to younger age groups. These fractures lead to severe dysfunction of the upper extremity in the near and distant periods after injury due to the development of contracture of the shoulder and joint long-term pain, which significantly complicates the patient’s life [8]. Choosing the proper treatment for these fractures in elderly and senile patients by conservative and operative methods (immobilization and early onset of movements, percutaneous osteosynthesis with needles, osteosynthesis with plates and pins, shoulder arthroplasty) still depends on the patient’s age, physical status, comorbidities [6, 9]. In old age, the course of the disease is complicated by age-related geriatric syndromes. Among them, sarcopenia and frailty are significantly associated with an increased frequency of falls [10]. In addition, these have adverse medical and social consequences, such as fear of recurrence, high-risk trauma [11, 12]. These diseases are independent indications for rehabilitation measures, primarily to improve motor control and balance (preventing falls and improving daily life activity). Nevertheless, their comorbidity and polymorbidity introduce into the recovery process individual-specific features directed at leveling the signs of each pathological condition and improving social functioning, which has become the direction and subject of our previous research [13, 14]. It substantiates the relevance of the presented work and determines its theoretical and practical value.

Our objective was to evaluate the corrective effect of a physical therapy program (based on functional training) on balance, upper limb function, activities of daily living, frailty, and quality of life of elderly patients with Parkinson’s disease following proximal humerus fractures.

## Material and Methods

A longitudinal prospective study involved 33 elderly patients with Parkinson’s disease and frailty following proximal humerus fractures. Subjects were divided into two groups by simple randomization. The control group (CG – 7 men and 9 women aged 68.1±3.1 years) underwent motor rehabilitation according to the principles recommended in the recovery period for proximal humerus fractures, using the general principles of classical kinesitherapy – correction of therapeutic exercises of muscular activity groups and other elements of the musculoskeletal system affected by trauma, neurological disorders, decreased overall motor activity etc [15, 16]. The treatment group (TG, 8 men, 9 women aged 64.9±1.8 years) consisted of patients who underwent a restorative treatment using a developed physical therapy program with an emphasis on active rehabilitation functional training, taking into account the comorbidity of Parkinson’s disease and frailty. Both groups of patients in rehabilitation also received massages of the upper extremity (10 sessions).

The developed physical therapy program lasted 3 months; corrective classes lasting 1 hour were held three times a week. The capacity level was gradually intensified by increasing the frequency, intensity, time, and type of exercise. The program was created taking into account the setting and consistent achievement of individual short- and long-term rehabilitation goals according to the International Classification of Functioning, Disability, and Health (ICF) [17]. Compensation for frailty (reduction of signs of sarcopenia and its impact on the quality of movements) in the tested program was carried out due to the direct effect of physical training on muscle tissue and dietary modification – normalization of protein, calcium, and vitamin D.

To prevent recurrences, the patient and their family members were trained in regards to safe movement (slowly, in low-heeled shoes, if necessary – with aids) and to create a safe environment at home (no carpets, sharp corners).

The effectiveness of the developed program was determined using tests that characterized the state of balance (risk of falling due to impaired motor control and deterioration of movement), upper limb function, the level of basic and instrumental functional activities, and reduced quality of life in dynamics before (pre-test) and after (post-test) implementation.

Patients were examined for SPPB, which is a marker of frailty and, at the same time, characterizes the state of equilibrium [18]. Testing was performed on the BBS, which is designed to detect changes in patient balance as an indicator of the risk of falling in unstable older people [19]. The relationship between the performance of household movements, taking into account the possibility of falling, was determined by FES-I [20]. Upper limb functionality was assessed using the standard DASH [21] scale and wrist dynamometer (the absolute value of which is also a criterion for frailty) [12]. Patient functional activities were defined as basic according to the ADL Index [22] and instrumental according to the IADL Scale [23]. Patients’ quality of life was assessed by PDQ-39 [24].

## Results

The initial examination of persons with frailty and Parkinson’s disease during the recovery period after proximal humerus fractures revealed low rates of muscle coordination, deterioration of the injured upper extremity, reduced basic functional and instrumental activity, and quality of life at high risk of re-fall. The results of all SPPB tests (balance, gait speed, chair stand), the evaluation of which directly points to the presence of frailty, were low in both groups, indicating a significant balance disorder, and therefore, a high risk of dependence on outside help, risks of falling. Failure to perform SPPB tests among elderly with frailty and Parkinson’s disease was associated with a high risk of falls, as confirmed by the BBS. The results of FES-I, which assesses the risk of falling due to the concern of performing some routine household manipulations, showed that, despite the long period after injury, examined patients feel uncomfortable in their normal household and social situations because they are afraid of falling again.

Functional impairment of the upper extremity due to prolonged immobilization combined with sarcopenia and extrapyramidal disorders in the examined patients differed significantly from the unaffected side. This was manifested by decreasing strength and impaired domestic functioning. The results obtained from determining the absolute strength of the wrist of an uninjured arm confirmed the presence of the frailty in the examined men and women. The strength of the injured arm in the examined patients was lower compared to the healthy arm by an average of 30%. Immobilization, muscle weakness, and tremor significantly impaired upper limb function in the examined patients, as reflected by DASH results.

Deterioration of postural stability and mobility in combination with dysfunction of the upper extremity on the background of extrapyramidal disorders led to a self-care deficit. Analysis of motor tasks based on the ADL Index showed that patients had a low ability to perform movements related to self-care at the initial examination. This was especially noticeable when performing complex coordination spatial movements – dressing, bathing, and going to the toilet. At the same time, such activities as eating and personal hygiene (due to tremor, hypokinesia) also deteriorated. Patients reported pelvic dysfunction due to the clinical course of Parkinson’s disease. According to the ADL Index, the overall level of baseline functional activity in both groups was assessed as a pronounced dependence. The results of the IADL Scale were also characterized by a low level (Table 1). To perform this type of activity, patients must have a sufficient level of fine motor skills, the ability to perform complex coordination movements of the upper extremities, and the absence of severe manifestations of dementia. The parameters obtained during the initial examination were distributed as follows. Patients showed satisfactory results in activities that did not require a high level of physical qualities – phone calls, medication, financial transactions (absence of moderate or severe dementia, which would reduce the cognitive ability to perform these actions, was a criterion for inclusion in the study). At the same time, the ability to perform such actions as shopping, cooking, and housekeeping deteriorated markedly, which manifested itself in a low total score on the scale. Consequently, this resulted in a decrease in the quality of life of the examined patients, based on the PDQ-39 questionnaire. The worst indicators were determined by the subscales “Mobility” (reflects the degree of limitation of physical activity) and “Activities of daily living” (characterizes the degree of limitation of self-care). The emotional well-being subscale, which shows the level of emotional background, was slightly better. Other criteria – “Stigma” (reflects the patient’s reaction to his disease and/or symptom when he is in society) and “Communication” (characterizes the level of communication restrictions) – were less impaired. The scales “Social support” (assesses the level of support for relatives), “Cognitions” (reflects the patient’s feeling of their cognitive functions), and “Bodily discomfort” (the patient’s feeling of discomfort in the body) were the least impaired.

**Table 1.** Dynamics of functional activity of elderly with frailty and Parkinson’s disease in the recovery period after proximal humerus fractures under the influence of physiotherapeutic functional training.

**Activities scores**	**Pre-test**					**Post-test**						
	**1**		**2**		**3**	**4**		**5**	**6**		**7**	**8**
	**Control group** **(n=16)**		**Treatment group** **(n=17)**		**p 1-2**	**Control group (n=16)**		**P 1-4**	**Treatment group (n=17)**		**p 2-6**	**P 4-6**
	**M**	**m**	**M**	**m**		**M**	**m**		**M**	**m**		
**ADL Index**	48.23	2.17	50.14	1.48	>0.05	57.45	1.29	<0.05	73.22	1.08	<0.05	<0.05
**IADL Scale**	4.03	0.06	4.11	0.09	>0.05	5.13	0.15	<0.05	6.21	0.12	<0.05	<0.05

According to the results of the initial survey, participants in the control and treatment groups did not significantly differ from each other (p>0.05). On re-examination, the parameters of the SPPB tests in both groups of patients and their associated frailty significantly improved relative to baseline. Progress in performing balance tests was 14% in CG and 27% in TG; walking speed – 11% and 42%, respectively; chair stand – 10% and 34%. The overall improvement in the total SPPB score was 12% in the CG and 35% in the TG (achievement by this group of the level of preasthenia in absolute digital level) (p<0.05 relative to the results of the initial survey).

Improved neuromuscular control and coordination in subjects reduced the risk of falling on the BBS. This was manifested by a significant improvement in the result (p<0.05) compared to the initial data – by 40% in CG and 75% in TG (reaching the digital level of “average risk of falling”).

Compared to initial data, the results obtained from FES-I confirmed greater confidence in their balance when performing household activities that could lead to a fall; the improvement in CG was 18%, TG – 53% (in both groups p<0.05 relative to the initial result).

Analysis of repeated wrist dynamometry, which characterizes the improvement of the functional reserve of the upper limb and characterizes the state of muscle tissue, showed the following facts: in both groups, there was a statistically significant difference compared to the initial result (p<0.05), increase in the parameters of the injured limb: in CG it was 12% in men and 14% in women, in TG – respectively 23% and 37% (*i.e.*, the best CG results). The increase in the strength of the uninjured hand reached a statistically significant level only in persons in TG – 11% in men and 16% in women (p<0.05 relative to baseline), and in CG, although it occurred in absolute numbers (6% in men, 5% in women), but not statistically significant (p>0.05). None of the groups achieved a result that would indicate the leveling of frailty signs in absolute indicators of wrist dynamometry in both men and women.

The benefits of functional physiotherapy training were identified when assessing the functioning of the upper extremity by DASH – improvement in CG was 25%, CO – 42% (p<0.05 relative to baseline).

IADL Scale re-examination results showed that patients with CG showed a statistically significant increase relative to the initial examination – 27% (p<0,05) (Table 1). On the other hand, TG showed an improvement not only in relation to the initial examination but also in relation to the CG indicator – 51% (p<0.05). Improving balance, postural stability, and functioning of the upper extremity improved the quality of life of the examined patients. According to the PDQ-39 questionnaire, the improvement compared to the initial result (p<0.05) was determined in both groups of “Mobility”, “Activities of daily living”, “Emotional well-being”, and in the CO – “Bodily discomfort”.

According to all studied indicators, patients of both groups showed statistically significantly better results compared to baseline (p<0.05), but individuals in TG showed better results than patients in CG (p<0.05). This indicates a higher efficiency of functional training directed at daily life activity than classical kinesitherapy, considering the comorbidity in the studied pathology.

## Discussion

It was proven that dopaminergic therapy plays a leading role in the treatment of Parkinson’s disease. However, drug treatment is predominantly the replacement therapy because it does not stop the progressive neurodegenerative process in the subcortical and other brain structures with dopaminergic mediation [1]. Therefore, as the duration of the disease increases, there is a steady rise in motor, autonomic and cognitive symptoms, even against a background of an adequate medical strategy, leading to severe deterioration in the quality of life and risks of concomitant pathological conditions in particular – falls [25, 26]. This raises the question of finding new corrective approaches for the recovery of patients with this disease. Recent experience shows that the most effective method is creating and implementing a multidisciplinary (drug and non-drug) approach for the rehabilitation of such patients. Such a treatment strategy significantly reduces the severity of the primary motor symptoms and non-motor disorders (cognitive and affective), leading to subjective and objective improvement in quality of life indicators [1].

Physical therapy is a leading non-drug remedy for Parkinson’s disease. Classes with a physical therapist are directed at strengthening the muscles of the extremities, and corset muscle correcting abnormal gait, which reduces muscle tone [27, 28], which is confirmed in our study. Occupational therapy, which is closely related to physical therapy, helps patients maintain independence in daily life, adapt to the disease, and become less dependent on its symptoms [26]. The disease frequently affects a person’s appearance: facial expression changes and/or their faces are significantly devoid of expression, the voice becomes quiet. As the disease progresses, the articulation suffers. Due to limited movement, it is difficult for patients to do their usual things. Working with a psychotherapist and being engaged in physical activity helps patients overcome the emotionally depressed state, teaches them to perceive themselves adequately, and reduces the external signs of the disease [26].

Numerous scientific studies have proven that kinesitherapy is a major factor in the rehabilitation of patients in the recovery period, which leads to regression of clinical manifestations, increases the range of motion in immobilized joints, promotes daily activity, and improves the quality of life of patients, enables patients to move independently and finally has a positive impact on patients’ mental health [29, 30]. In particular, this applies to the proximal humerus fractures – a trauma associated with osteoporosis in the elderly, which causes their disability [7, 8]. At the same time, it is recommended to use most kinesitherapy techniques as training and recovery designed for individual muscle groups, without linking the techniques to movements and activities of daily living [15, 16] that can delay and reduce a therapeutic effect, which is confirmed by the results of our study.

Age-related conditions such as geriatric syndromes, among which frailty has one of the important places, require physical exercises to correct them; the effectiveness of exercises is also established when eliminating the phenomena of sarcopenia [10]. The development of those conditions is accompanied by a decrease in physical and functional activity, in adaptive and restorative reserves of the body, and an increase in the risk of negative consequences – hospitalization, functional deficits, physical limitations, falls, and fractures [11, 31]. Consequently, the main objectives of physical therapy among elderly, who suffer from comorbid and polymorbid pathological conditions are to restore impaired motor function, maintain optimal mobility of patients for as long as possible, improve their quality of life by maintaining or increasing patients’ independence, safety and well-being [3]. In our program, these objectives were achieved by preventing falls and, accordingly, a sedentary lifestyle, improving daily activity, and reducing limitations in household activities, which ultimately led to a statistically significant improvement in the quality of life of the elderly with combined pathological conditions.

## Conclusion

The problem of restoring the health and quality of life of elderly patients with comorbid and polymorbid pathological conditions by non-drug therapy, primarily through physical activity, is an urgent problem of rehabilitation medicine and society. Elderly patients with Parkinson’s disease and frailty after proximal humerus fractures have a high risk of falls, upper limb dysfunction, impaired domestic and social functioning, a low level of activities of daily living, the deterioration of quality of life, as evidenced by the results of the SPPB, BBS, wrist dynamometry, DASH, FES-I, ADL Index, IADL Scale, PDQ-39. Elderly patients need to take part in individualized physical therapy programs, considering the specifics of each disease, directed at restoring self-care and improving the quality of life, which increases the overall effectiveness of rehabilitation and, consequently, may reduce the burden on social and medical services who take care of the problems of older people.

## Acknowledgments

### Conflicts of interest

The authors declare no conflict of interest.

### Ethical approval

This study was approved by the Ethics Committee of the HSEEU Vasyl Stefanyk Precarpathian National University, Ivano-Frankivs’k, Ukraine (approval ID: 02-15.09.2021).

### Consent to participate

Written informed consent was obtained from the patients.

### Data availability

Further data is available from the corresponding author on reasonable request.

### Sources of funding

This study was funded by the Vasyl Stefanyk Precarpathian National University.

### Authorship

HB and OZ contributed to conceptualizing the study. HB and PY contributed to the methodology. HR contributed to writing the original draft. KH contributed to editing the manuscript. LB, VL contributed to data collection. SL, HO contributed to interpreting the results and worked on the manuscript. ZO, BT contributed to data curation. LE contributed to data analysis.
